# 

**Published:** 1984-10

**Authors:** 


OCTOBER 1984

VOLUME 99 (iv)

NUMBER 372

Published Quarterly

Annual Subscription £8 Post Free

### BRISTOL MEDICO-CHIRURGICAL JOURNAL

Established 1883

### BRISTOL MEDICO-CHIRURGICAL JOURNAL

Incorporating the Medical Journal of the South West

### Editorial Committee

**EDITOR**

*Mr. M. G. Wilson*
*40 Coombe Lane, Bristol BS9 2BJ*

MEMBERS

*Dr. M. B. Lennard*
*Dr. D. W. Wright*
*The Hon. Treasurer of the Society*
*The Hon. Secretary of the Society*

SECRETARY

*Mr. B. P. Jones, The Library, Medical School, University Walk, Bristol BS8 1TD*

### Correspondents

*Dr. J. D. Davies*
*Dr. J. Jancar*
*Dr. H. G. Mather*
*Professor G. M. Stirrat*
*Dr. J. L. G. Thomson*
*Dr. M. J. Whitfield*
*Professor R. C. N. Williamson*
*Dr. D. W. Wright*

### Bristol Medico-Chirurgical Society

PRESIDENT

*Dr I. S. Bailey*

PRESIDENT ELECT

*Dr J. Jancar*

HONORARY SECRETARY

*Dr. H. M. Norman*
*Hillside, Vicarage Lane, Barrow Gurney*

HONORARY TREASURER

*Dr. J. B. Penry.*
*Southmead Hospital, Bristol BS10 5NB*

The Society was founded in 1874. In 1883 publication of the Journal began and has continued quarterly ever since then. During the years 1949 to 1962 it appeared under the title of 'The Medical Journal of the South West'.

### Notice to Contributors

*The Journal is especially concerned to provide a place for the recording of medical thought, interests and practice in and around Bristol. It therefore welcomes articles that, as well as being of immediate interest to members, will document the local and contemporary medical scene for our successors.*

*Original articles are preferred in the form proposed by the International Committee of Medical Editors (The Vancouver System) and published as an article in the British Medical Journal in 1979 (Br. Med. J.* **1***, 532–535). This has been reprinted as a pamphlet and may be obtained from the Editor of the B.M.J., B.M.A. House, Tavistock Square, London WC1H 9JR (enclose 50p).*

*Also welcome are letters to the Editor, Book Reviews, news of important appointments and events, etc.*

Printed in Great Britain by John Wright & Sons (Printing) Ltd, at The Stonebridge Press, Bristol

### University of Bristol Faculty of Medicine

### THE DEGREE OF DOCTOR OF MEDICINE

**June 1984**

David Francis LEVINE
Demetrios Athanasiou VASSILOPOULOS

**October 1984**

Peter Michael GOUGH
Geoffrey Henry HUTCHINSON

### THE DEGREE OF MASTER OF SCIENCE IN MEDICAL SCIENCES

**June 1984**

Edmundo Carvalho MAUAD

**October 1984**

Yousef Elia Yousef ABOUSH
Ann Frances BAGSHAW
Kholood Najeeb SAFAR

### THE DEGREE OF DOCTOR OF PHILOSOPHY

**June 1984**

Jill Ellen ARTHUR
Rochelle Ann Steiner GIBBONS
Loraine Elizabeth GODDARD
Joan Lamise KEIRBY
Terry MORGANS
Raymundo RODRIGUEZ DE LARA
Phillip Rodney WIDDERS

**October 1984**

Sarah Louise EWERS
Peter Walter MADDEN
Caroline Jean OWENS
Masoud TESHFAM

### FINAL EXAMINATION FOR THE DEGREES OF M.B., Ch.B. June 1984

PASS

*With Honours*

Susan Ruth BAXTER – with Distinction in Obstetrics and Gynaecology
Allan DAWSON – with Distinction in Surgery
Amanda Suzanne HIGGINS – with Distinction in Mental Health
Richard Derek IGGO – with Distinction in Medicine
Ayo Elizabeth Salome PALMER – with Distinction in Child Health
David Michael WARD – with Distinction in Child Health

*Pass*

Victoria Yvonne ALLISON
Jonathan St. Clair ANDERSON – with Distinction in Mental Health and in Community Medicine
Philip Adriaan Gerard BAKKER
Karen Anne BARBACK
Maria Louise BARNARD
Susan Jean BENBOW
Jane Mary BENNETT
Andrea Donna Mary BLIGH
Philip Anthony BLOOM
David John Wilton BOOTH
Helen Finch BOWDEN
Claire Elizabeth BROOKS
Isobel Clare BROWN
Lucinda Jane CARR
Bernadette Fung Mei CHOW
Chuk Yin Lisette CHUNG NGAN HING
Bernadette Bridget CLARKE – with Distinctions in Obstetrics and Gynaecology and in Community Health
John COLLINGE
Rebecca Jane COLLIS
Neil Christopher COOPER
Susan Nicola Allison COX
Dudley Robert DARVILL
Jeremy Paul DIAMOND
Catherine Laura DICK – with Distinctions in Mental Health and in Community Health
Jennifer DIXON
Kieron Louis DONOVAN
Christopher John DUCKER
Vivien Ann EDWARDS
Geraldine Mary ELCOMBE
Kenneth Brian FRANKLIN
Nicola GAINSBOROUGH
Mary Lynn GARDNER
Fiona Alison Murray GIBSON
Alison Margaret GLAISTER
Lawrence Charles GOLDBERG
Timothy Howard GOULD

David John GUNNELL
Mitra Dave GUNPUT
Christine Maria HABGOOD
Peter Nicholas HARBORD
Neil Peter HARRIS
James HILL
Jennifer Nan HODGSON
Gillian Margaret Claire HOLDSWORTH
Robert Patrick Hunter HOLMES
Simon Peter HORSLEN
Susan Mary HUDSON
Nigel JONES
Peter John KIRKPATRICK
Michael Gareth KNOWLES
John Francis LEAHY
Joanne Melissa LEE
Claire MACGREGOR
Ian Norman Clowes MACLEOD
Anne Louise MARSHALL
Charlotte Elizabeth Orgill MASSEY
Thomas Gray MAXWELL
Alina Phoebe MCGARRIGLE
Jeanette McLOUGHLIN
Neeraj Kumar MEDIRATTA
Balvinder Singh MEHAT
Helen Clare MEYRICK
Peter Stephen MILNE
Joyce Marie MULVEY
Clare Siobhan MURPHY
Paul Gerard MURPHY – with Distinction in Pathology
William James Crawford MURPHY
Andrew William MURRISON
Claire Theresa NESLING
Bobby Kin Wah NG
Julie Anne NICHOLLS
James Alan Ramsay NICOLL
Iain Samuel NOBLE
Francis Paul NOLAN
Jane Tracy O'GRADY
Sean William O'KELLY
Edward James Hoblyn OLIVER
Philip Peter OLIVER
Richard Gek Beng OOI
Gerwyn OWEN
Jane Elizabeth Victoria PARKES
Guy Michael PATRICK
Marc Xavier PELLING
Rohanta Kumar PERERA
Catherine Mary PINCHES
Ian John PORTER
Geoffrey James RAINE
Sharon Jane RICHARDSON
Susan Elizabeth Kim ROBERTS
Martin Gerard RONCHETTI
Mary Ann RUTHERFORD – with Distinctions in Child Health and in Mental Health
Ian George RYDER
Jane Elizabeth SANSOM
Philip Martyn John SCOTT
Susan Mary SHARPLES
Ehoud SHMUELI
Mark Kenneth SIMMONDS
Tajinder SINGH
Christopher Michael SMITH
Kathryn Jane SMITH
Peter Stanley TALBOT
Solomon TESFAYE – with Distinction in Mental Health
Chandraraj Jeyapalan THAMOTHERAM
Rosemary Ann Natalie THOMAS
Neil James TODD – with Distinction in Pathology
Darryl Russell WATTS
Michael John WESTON
Anna Marie WHEATLEY
Richard John WHEATLEY
Adela Mary WILLIAMS
Ruth Margaret WILLIS
Neil John WRIGHT
Philip Arthur Lawson WYLIE

